# Hepatic damage caused by long-term high cholesterol intake induces a dysfunctional restorative macrophage population in experimental NASH

**DOI:** 10.3389/fimmu.2022.968366

**Published:** 2022-09-08

**Authors:** Ana C. Maretti-Mira, Matthew P. Salomon, Angela M. Hsu, Gary C. Kanel, Lucy Golden-Mason

**Affiliations:** ^1^ USC Research Center for Liver Disease, Keck School of Medicine, University of Southern California, Los Angeles, CA, United States; ^2^ Division of Gastrointestinal and Liver Disease, Department of Medicine, Keck School of Medicine, University of Southern California, Los Angeles, CA, United States; ^3^ Department of Pathology, Keck School of Medicine, University of Southern California, Los Angeles, CA, United States

**Keywords:** nonalcoholic fatty liver disease (NAFLD), Kupffer cells, tissue macrophages, RNAseq, innate immunity, cholesterol

## Abstract

Excessive dietary cholesterol is preferentially stored in the liver, favoring the development of nonalcoholic steatohepatitis (NASH), characterized by progressive hepatic inflammation and fibrosis. Emerging evidence indicates a critical contribution of hepatic macrophages to NASH severity. However, the impact of cholesterol on these cells in the setting of NASH remains elusive. Here, we demonstrate that the dietary cholesterol content directly affects hepatic macrophage global gene expression. Our findings suggest that the modifications triggered by prolonged high cholesterol intake induce long-lasting hepatic damage and support the expansion of a dysfunctional pro-fibrotic restorative macrophage population even after cholesterol reduction. The present work expands the understanding of the modulatory effects of cholesterol on innate immune cell transcriptome and may help identify novel therapeutic targets for NASH intervention.

## Introduction

The liver is a crucial metabolic organ for lipid biosynthesis, processing, and elimination. Abnormal hepatic lipid accumulation is a hallmark of steatosis, a conditional part of the nonalcoholic fatty liver disease (NAFLD) spectrum. These lipids typically derive from diet, *de novo* lipogenesis, or adipose tissue lipolysis (1). Hepatic steatosis is frequently benign but can progress to nonalcoholic steatohepatitis (NASH), characterized by hepatic cellular injury and necroinflammation (2). NASH may progress to cirrhosis, end-stage liver failure, and hepatocellular carcinoma, representing the most common reason for terminal hepatic failure in western societies (3).

A significant change in lipid metabolism is also evident in NAFLD. A marked growth in free cholesterol (FC) levels occurs during the progression of steatosis to NASH, and total plasma cholesterol correlates to the presence of hepatic inflammation (4, 5). Increased cholesterol synthesis combined with a decrease in cholesterol elimination or the excessive intake of dietary cholesterol results in FC accumulation in the liver (6, 7). FC accumulation impacts several hepatic cells. FC accumulation in hepatocytes often triggers mitochondrial oxidative stress, sensitizes hepatocytes to pro-inflammatory cytokines, and later leads to cell death (8, 9). Hepatic stellate cells loaded with FC become sensitive to transforming growth factor (TGF) β-induced activation, which accelerates liver fibrosis (10), while FC exposure and accumulation in liver resident macrophages, termed Kupffer cells (KCs), contribute to liver inflammation (11).

KCs are part of a large family of innate immune effector cells known as macrophages. Hepatic macrophages fall into two categories: resident macrophages, the KCs, and infiltrating macrophages (IMs), derived from circulating monocytes that arrive at the liver during inflammation. Hepatic macrophages represent the first line of defense against products coming from the gastrointestinal tract and can play both pro- and anti-inflammatory roles in chronic liver diseases and directly contribute to fibrosis progression and resolution (12). Expansion of hepatic macrophages is a marker for NAFLD severity, and, in general, their participation is described as pro-inflammatory (13–15). Hepatic macrophage depletion prevents steatosis development and decreases hepatic levels of fibrosis (16). However, macrophage depletion during the NASH recovery phase accentuates liver fibrosis, suggesting a broader involvement of macrophages in liver homeostasis during NAFLD (14).

Although several studies support the participation of hepatic macrophages in NAFLD progression, the impact of dietary cholesterol on hepatic macrophage transcriptome and function in the setting of NASH remains elusive. Animal models of NASH based on long-term high-fat and high-fructose diets display the highest similarity to human NAFLD, not only phenotypically but also at the transcriptomic level (17). Therefore, we have selected an established prolonged fructose, palmitate, cholesterol, and trans-fat (FPC) diet to develop murine NASH and fed mice with different contents of cholesterol in two stages of our dietary intervention, starting with medium (0.2%) or high (1.25%) cholesterol content, and later reducing cholesterol to low levels (0.05%) (18). Our findings suggest that a decrease in cholesterol intake silences the inflammatory signal detected in macrophages from mice initially fed with medium cholesterol FPC diet. However, the effect of a long-term diet fueled by high cholesterol content is more harmful to the liver homeostasis since the hepatic macrophages do not completely deactivate after cholesterol reduction and further display a dysfunctional restorative macrophage phenotype.

## Materials and methods

### Murine model of diet-induced NASH

To evaluate the contribution of dietary cholesterol to NASH progression, we selected the well-established Fructose, Palmitate, Cholesterol, and Trans-Fat (FPC) diet (Envigo) to induce NASH as this model recapitulates human disease (17, 18). Seven-week-old male wild type C57BL/6J mice (Jackson Laboratory) were allowed to acclimate to housing in our facility for two weeks before the dietary intervention, which was subdivided into two phases: 1) Progression: mice received the FPC diet for 20 weeks and were divided into three groups according to the dietary cholesterol content - low (FPC + 0.05% cholesterol), medium (FPC + 0.2% cholesterol) and high (FPC + 1.25% cholesterol). 2) Regression: Cholesterol was reduced to 0.05% in all the FPC diet subgroups for additional ten weeks. The animal study was reviewed and approved by the University of Southern California Institutional Animal Care and Use Committee.

### Liver non-parenchymal cell isolation

Hepatic non-parenchymal cells (NPCs) were isolated using liver perfusion. Livers were perfused with calcium/magnesium-free HBSS containing 3mg/mL of Liberase™ (Roche), excised, and then mechanically dissociated in calcium/magnesium-free HBSS containing 0.5% BSA and 2mM EDTA. The cell suspension was centrifuged at 20 x g for 2 minutes to remove hepatocytes and then transferred to a new tube and centrifuged at 365 x g for 8 minutes. Red blood cells were removed by lysis (RBC lysis buffer - BD Pharmlyse), and NPCs were isolated by density gradient centrifugation with 20% OptiPrep™ (StemCell Technologies). The NPC layer was collected and stained for cell sorting.

### Hepatic macrophage sorting

To identify hepatic macrophages, we selected several markers that could identify hepatic macrophages with different phenotypes (19–21). We stained NPCs with anti-mouse CD45 (BD Horizon; 30-F11), MertK (eBio; DS5MMER), CD64 (BioLegend; X54-5/7.1), F4/80 (eBio; BM8), and CD11b (eBio; M1/70) antibodies. Cell incubation took place on ice in the dark for 25 minutes. Sorting was performed on a BD FACSAria™ Fusion Flow Cytometer, with 100µm nozzle, into 90% FBS media at 4˚C. After sorting, we checked population purity and centrifuged cells at 400 x g for 5min at 4°C. Cells were resuspended in RLT buffer (Qiagen) containing 2-mercaptoethanol, passed through QIAshredder (Qiagen), and stored at -80°C for subsequent processing.

### Macrophage bulk mRNAseq

Macrophage RNA was isolated using the RNeasy kit (Qiagen) following the manufacturer’s protocol. RNA quality and concentration were assessed by the 2100 Expert Bioanalyzer System (Agilent), using the RNA 6000 Pico Kit (Agilent). Transcriptome RNA sequencing was performed at the Norris Cancer Molecular Genomics Core. Library quality control was perform using Agilent BioAnalyzer 2100 and libraries were simultaneously prepared using Kapa mRNA Hyper kit (cat#08098123702, Roche Diagnostics) following the manufacturer’s protocol. RNAseq libraries were sequenced on the Illumina Nextseq500 platform at a read length of 2x75 at 25 million reads per sample.

Raw sequencing reads were checked for overall quality and adapter contamination using FastQC (https://www.bioinformatics.babraham.ac.uk/projects/fastqc/) and trimmed using Trim Galore (https://www.bioinformatics.babraham.ac.uk/projects/trim_galore/) prior to downstream analysis. Reads were then used to quantifying transcript abundances with Salmon (22) using the GENCODE version M25 mouse reference. The resulting transcript abundances were summarized to gene level counts using functions in the Bioconductor package tximport (23). Significantly differentially expressed genes were identified using the Bioconductor package DESeq2 (24) with a significance threshold of FDR < 0.1. Volcano plots were generated using the Enhanced Volcano Bioconductor package (https://github.com/kevinblighe/EnhancedVolcano). Ingenuity Pathway Analysis (IPA) software (v01-20-04, Qiagen) was used to determine the hepatic biological processes altered by cholesterol intake level (25). Gene Set Enrichment analysis (GSEA) software v4.1.0 was used to identify relevant pathways and biological processes (26). The Gene Ontology Resource platform was used to identify the biological processes triggered by the commonly expressed genes during the progression phase of the dietary intervention (27).

### Hepatic inflammation and fibrosis histological assessment

Liver fragments from each lobe were kept in 10% buffered neutral formalin overnight and dehydrated in 70% ethanol at 4°C. Fragments were then embedded in paraffin and cut into sections of 5µm thickness, deparaffinized, and hydrated. The serial sections were then stained for H&E and Sirius Red as per standard protocols at the USC Research Center for Liver Diseases (RCLD) Liver Histology Core.

Liver biopsies from 4 lobules from each mouse used in this study was evaluated and graded histologically as follows: Steatosis grades: “0” =none, “<1” = less than 5% of hepatocytes, “1” = 5-25%, “2” = 26-50%, “3” = 51-75%, “4” = >75%; Lobular inflammation grades (20x field): “0” = none, “1” = < 2 per field, “2” = 2-4 per field, “3” = > 4 per field; Fibrosis grades: “0” = None, “1” = Perisinusoidal or periportal, “2” = Perisinusoidal and portal/periportal, “3” = Bridging fibrosis, “4” = Cirrhosis. The scoring was performed by a pathologist (G.K) in coded fashion without knowledge of the treatment.

### Statistical analyses

All statistical analyses, graphs, and heatmaps were generated using GraphPad Prism version 9 for macOS (GraphPad Software, www.graphpad.com). We used the non-parametrical Kruskal-Wallis test to evaluate hepatic inflammation and fibrosis scores.

## Results

### High dietary cholesterol intake exacerbates hepatic inflammation and fibrosis

**Figure 1:**
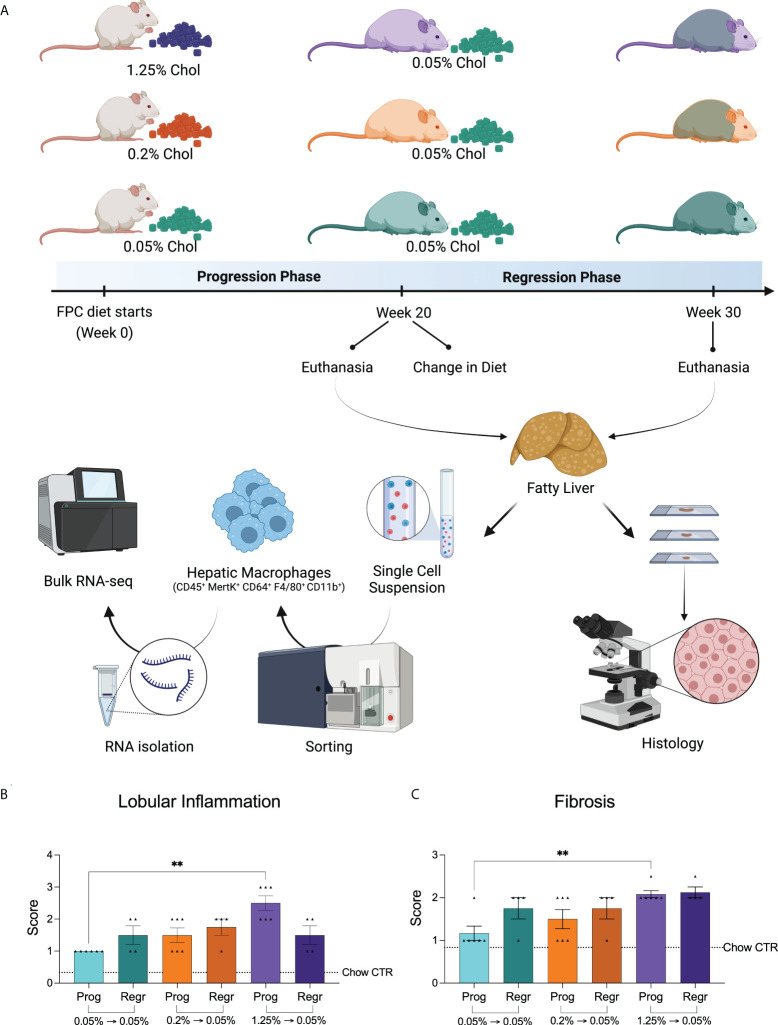
Experimental design and dietary cholesterol impact on murine NASH model. **(A)** For our NASH model, we selected the Fructose, Palmitate, Cholesterol, and Trans-Fat (FPC) diet. We evaluated the mice in two distinct phases. During the first 20 weeks (progression phase), we fed male C57BL/6J mice with the FPC diet containing different concentrations of cholesterol: 1.25% (high, purple), 0.2% (medium, orange), and 0.05% (low, green). At week 20, we reduced the dietary cholesterol intake for the 1.25% and 0.2% groups, keeping all mice on the FPC diet with 0.05% cholesterol for additional ten weeks (regression phase). At the end of each phase, we harvested mice livers, sorted hepatic macrophages, and processed them for RNA-seq analysis. NASH was assessed on the liver specimens based on histologic findings (Created with BioRender.com). **(B)** Lobular inflammation and Fibrosis **(C)** were exacerbated in mice fed with high cholesterol FPC diet during the progression phase (n = 6 for progression and n = 4 for regression phases). Statistical test used: Kruskal-Wallis, **P value < 0.01. Prog, Progression phase; Regr, Regression phase; CTR, control.

To evaluate the contribution of dietary cholesterol to the progression of nonalcoholic steatohepatitis (NASH) induced by high-fat and high-fructose diet, we used the FPC diet as an established model mimicking human NASH (18). We combined the FPC diet with different cholesterol concentrations into two distinct phases. In the first phase (progression), mice were fed the FPC diet for 20 weeks with high (1.25%), medium (0.2%), or low (0.05%) cholesterol. In the second phase (regression), we reduced the cholesterol content of all FPC diets to 0.05% and fed all the groups for a further ten weeks. We euthanized six mice from each group at the end of the progression phase and four at the end of the regression phase, harvesting their livers to evaluate steatosis, inflammation, and fibrosis. We also isolated hepatic macrophages for transcriptomic analysis (**Figure 1A**).

As expected, all FPC diet groups showed advanced steatosis (grade 4, **Supplementary Figure 1A**) without differences related to cholesterol intake. We observed a significant increase in lobular inflammation and fibrosis in the livers of mice fed with high cholesterol during the progression phase (**Figures 1B, C**; **Supplementary Figures 1B, C**). While inflammation slightly improved after cholesterol reduction (not significantly), fibrosis remained unchanged. In fact, the fibrosis scores of all groups became very similar by the end of the regression phase (**Figure 1C**).

Our findings suggest that high cholesterol intake combined with a high-fat and high-fructose diet aggravates hepatic inflammation and accelerates fibrosis.

### Cholesterol intake affects hepatic macrophages transcriptome

**Figure 2:**
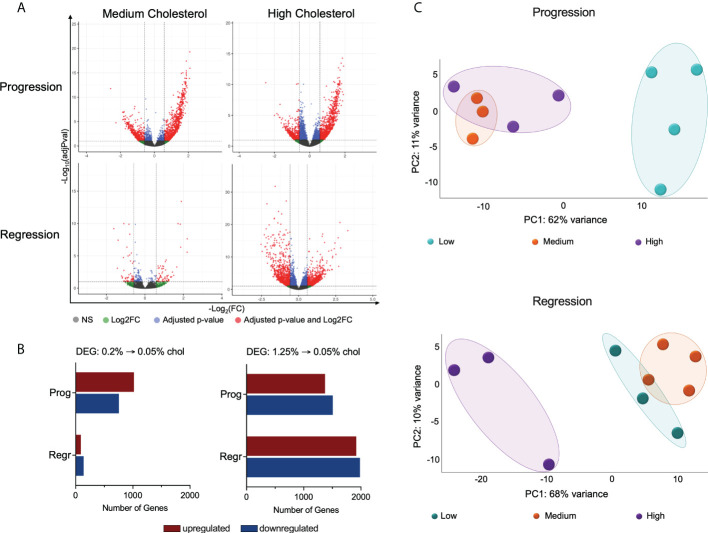
High cholesterol intake changes the global gene expression of hepatic macrophages. **(A)** Volcano plots show the hepatic macrophages’ differentially expressed genes (DEGs) in the different dietary groups, emphasizing the cholesterol effect. DEG lists were acquired by normalizing the transcriptome from the high and medium cholesterol groups using the age-matched low cholesterol group. **(B)** Comparing the medium to the low cholesterol group, we found 1,774 genes modified during the progression phase (1,019 up and 755 down) and 229 genes in the regression phase (90 up and 139 down). The comparison between the high to the low cholesterol group revealed 2,880 genes differentially expressed during the progression phase (1,373 up and 1,507 down), and 3,902 DE genes in the regression phase (1,918 up and 1,984 down). **(C)** PCA plots clearly show that during the progression phase, the medium cholesterol (n = 3) and the high cholesterol (n = 3) groups differed from the low cholesterol group (n = 4) and that after cholesterol reduction (regression phase), the medium cholesterol group (n = 4) became comparable to the low cholesterol group (n = 3), while the high cholesterol group (n = 3) maintain the original difference. NS, non-significant; Log2FC, log2-foldchange based on low cholesterol age-matched group; Prog, Progression phase; Regr, Regression phase.

Considering the significance of hepatic macrophages for NASH development, we evaluated the impact of dietary cholesterol on total hepatic macrophage transcriptome in a high-fat and high-fructose diet background. For this purpose, we sorted hepatic macrophages from the non-parenchymal cells obtained from mice livers, acquiring an average of 400,000 hepatic macrophages from each mouse. Cells were immediately preserved in RLT lysis buffer (Qiagen™) and later processed for RNAseq. Our findings revealed that cholesterol addition to the FPC diet significantly changed hepatic macrophage global gene expression (**Figures 2A, B**). We compared the medium and high cholesterol groups to the low cholesterol group in the different dietary phases to identify genes regulated by cholesterol. In macrophages from mice fed with medium cholesterol, we identified 1,774 differentially expressed (DE) genes (1,019 up and 755 down) during the progression phase and 229 genes (90 up and 139 down) during the regression phase. The most remarkable changes were observed in macrophages from the high cholesterol group, with 2,880 DE genes (1,373 up and 1,507 down) during the progression phase and 3,902 DE genes after cholesterol reduction (1,918 up and 1,984 down). Principal component analysis (PCA) indicated that macrophages’ transcriptomes from mice fed with high and medium cholesterol are similar during the progression phase, fully separating from the low cholesterol group. However, during the regression phase, macrophages from the medium cholesterol group normalized their gene expression, becoming similar to macrophages from the low cholesterol group (**Figure 2C**). These results suggested that the transcriptomic modifications triggered by a high cholesterol diet are more robust and not as easily reversed as those resulting from medium cholesterol intake.

### Identification of genes modulated by dietary cholesterol in hepatic macrophages

**Figure 3:**
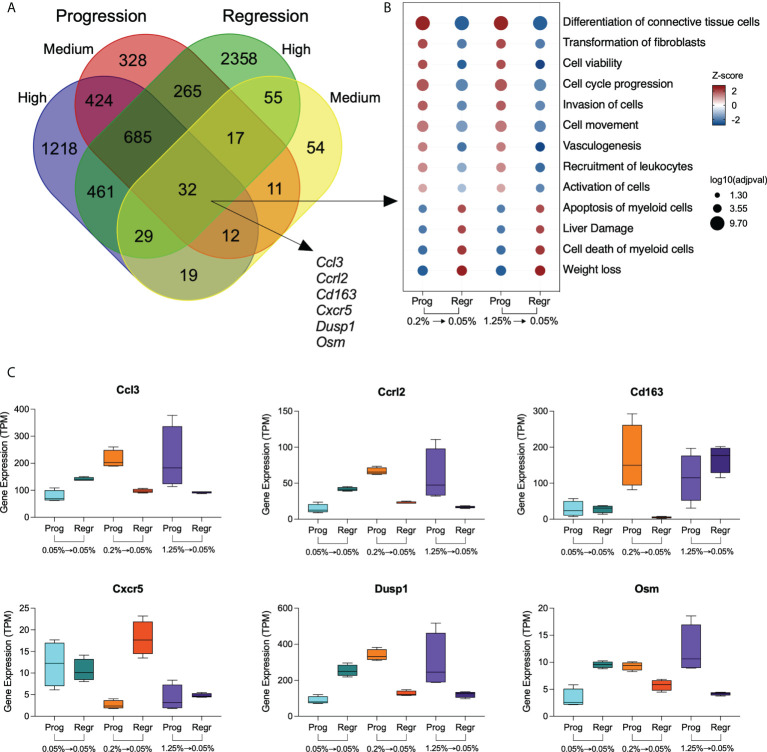
Genes modulated by dietary cholesterol in hepatic macrophages. **(A)** Venn diagram shows the overlapping differentially expressed genes among the comparison groups. The high and medium cholesterol groups were compared to the low cholesterol group during progression (Prog) and regression (Regr) phases. Thirty-two genes were commonly expressed by the four comparison groups. **(B)** Bubble plot shows the biological processes derived from the 32 genes commonly expressed genes by the four groups. Analysis performed using Ingenuity Pathway Analysis (IPA). **(C)** Expression of six genes with immunological significance were highlighted are shown in all six dietary groups included in this study. Prog, Progression phase; Regr, Regression phase.

We analyzed the relationship among the DE genes from the high and medium cholesterol groups in both dietary phases and identified 32 genes commonly expressed, which were also strongly connected as a network (**Figure 3A**; **Supplementary Figure 2**; **Supplementary Table 1**). The biological processes enriched by those genes were related to differentiation and transformation of connective tissue cells, cell cycle, viability and activation, cell movement, and vasculogenesis. These processes were stimulated by cholesterol intake and inhibited by cholesterol reduction. Other processes also enriched by these genes were cell death, liver damage, and weight loss, downregulated by cholesterol ingestion and upregulated by cholesterol intake reduction (**Figure 3B**).

We highlighted genes with immune relevance in **Figure 3C**. Cholesterol intake upregulates the expression of *Ccl3, Cclr2, Dusp1, Osm*, and *Cd163*. Cholesterol reduction normalized the expression of *Ccl3, Cclr2, Dusp1*, and *Osm* in both medium and high cholesterol groups, while *Cd163* expression only decreased in the medium cholesterol macrophages. Conversely, *Cxcr5* expression was downregulated by cholesterol intake, recovering its levels after cholesterol reduction in the medium but not in the high cholesterol group.

**Figure 4:**
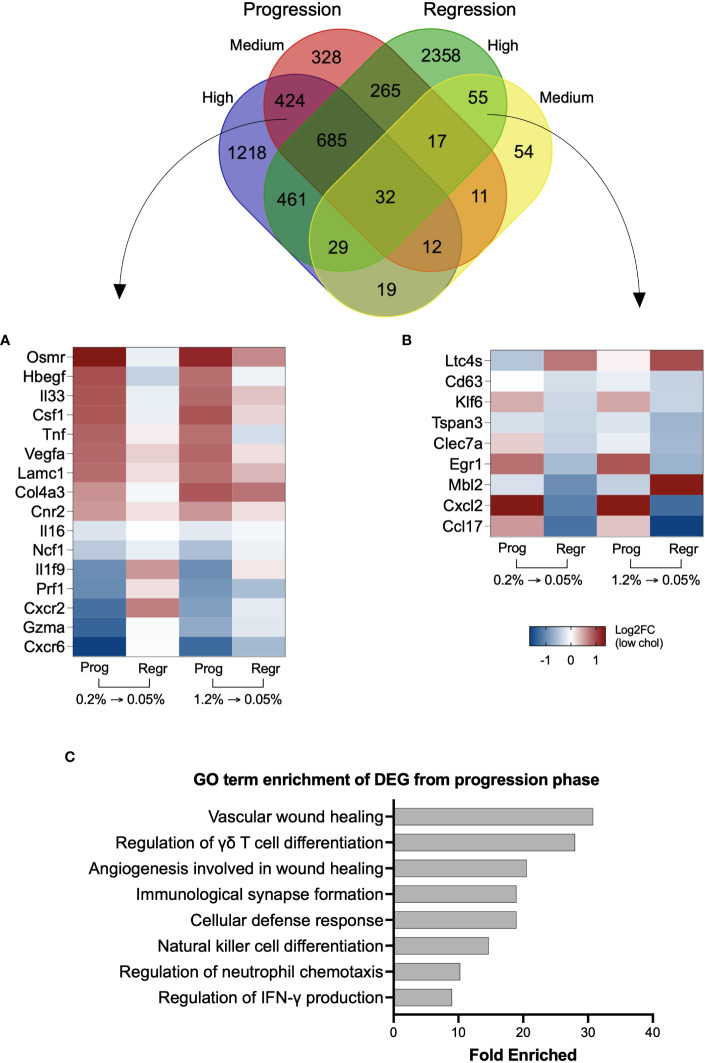
Genes uniquely modified by inclusion or reduction of dietary cholesterol can contribute to NASH. Venn diagram with the overlapping differentially expressed genes from the four groups. High and medium cholesterol groups commonly expressed 424 genes during the progression phase, while only 55 genes uniquely were commonly found during the regression phase. **(A)** Heatmap with 16 genes from the 424 genes upregulated by cholesterol introduction. The selected genes are previously correlated to NAFLD severity. Overall, these genes followed a similar expression pattern during the progression phase of medium and high cholesterol groups, with reversal of expression pattern on cholesterol reduction. **(B)** Heatmap showing 9 of the 55 genes commonly expressed in medium and high cholesterol groups after cholesterol reduction. The genes of interest are involved in NASH inflammation. **(C)** Gene Ontology (GO) terms enrichment using the differentially expressed genes (DEG) induced by cholesterol during the progression phase. Most of the biological functions were related to immune response. No significant results were found using the 55 genes commonly modified during the regression phase. Log2FC, log2-foldchange based on low cholesterol age-matched group. Prog, Progression phase; Regr, Regression phase.

We found 424 genes uniquely modified by cholesterol intake, regardless of its concentration, which included *Cnr2, Col4a3, Csf1, Cxcr2, Cxcr6, Gzma, Hbegf, Il16, Il1f9, Il33, Lamc1, Ncf1*, *Osmr, Prf1, Tnf*, and *Vegfa* (**Figure 4A** and **Supplementary Table 1**). Cholesterol reduction exclusively affected the expression of 55 genes, including *Ccl17, Cxcl12, Cd63, Clec7a, Tspan3, Erg1, Klf6, Ltc4s*, and *Mbl2* (**Figure 4B** and **Supplementary Table 1**). We applied the Gene Ontology enrichment test to these two sets of genes and observed that only genes from the progression phase could enrich relevant biological processes, such as wound healing, cytotoxic cell differentiation, neutrophil chemotaxis, and immune response (**Figure 4C**).

### Dietary cholesterol induces macrophage genes related to extracellular matrix organization, inflammation and affects macrophage polarization

**Figure 5:**
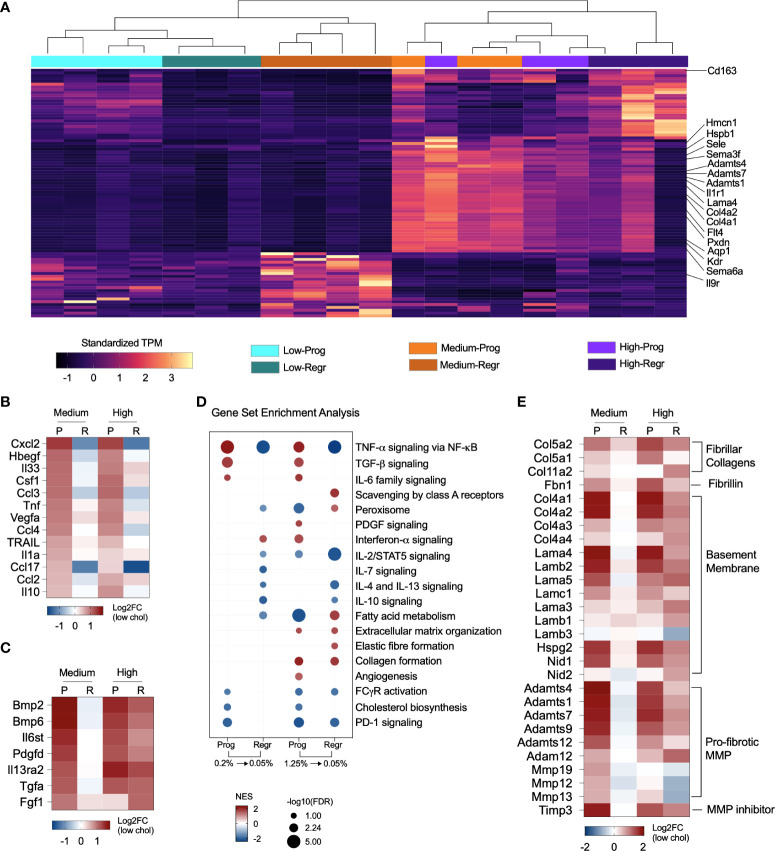
Cholesterol triggers expression of pro-fibrotic and pro-inflammatory genes. **(A)** Heatmap shows the standardized gene expression values of the 25 top upregulated genes found in each group. The highlighted genes commonly upregulated in the medium and high cholesterol groups during progression phase (Prog) and in the high cholesterol groups during regression phase (Regr) are mainly related to extracellular matrix organization, angiogenesis, and cell migration. **(B)** Expression of several cytokines relevant to NASH are affected by dietary cholesterol. In general, these cytokines were upregulated due to increased cholesterol dietary content and show an attenuated expression pattern after cholesterol reduction. **(C)** Cytokines upregulated during progression phase in both cholesterol groups and that maintained high expression in the high cholesterol groups after cholesterol reduction. Genes normalized in the medium cholesterol group during regression phase. **(D)** Gene Set Enrichment Analysis results, based on Hallmark and Reactome databases, highlighted the upregulation of TNF-α, TGF-β, and IL-6 signaling pathways during the progression phase of both cholesterol groups with subsequent downregulation/normalization after cholesterol reduction. The pathways for IL-10, IL-4/IL-13 signaling were downregulated by cholesterol reduction in both groups. Lipid metabolism, FC-γ receptor activation and PD-1 signaling are inhibited in both groups in the progression phase. The high cholesterol group showed continuous upregulation of collagen formation in both progression and regression phases. **(E)** Heatmap showing the fold changes in the expression of fibrillar collagens, fibrillin, type IV collagen and laminin, other basement membrane components, and pro-fibrotic proteases. Gene expression was upregulated in medium and high cholesterol groups during progression phase and stayed elevated in the high cholesterol after cholesterol reduction. P or Prog, Progression phase; R or Regr, Regression phase; Log2FC, log2-foldchange based on low cholesterol age-matched group.

We identified the top 25 upregulated DE genes from the high and medium cholesterol groups during both the progression and regression phases, clustered the gene expression values of all six groups, and observed that the samples clustered into two main branches (**Figure 5A** and **Supplementary Table 2**). The first branch grouped all samples from the low cholesterol groups and the samples of the medium cholesterol group during the regression phase. The other branch comprised samples from the medium cholesterol group during the progression phase and all the samples from the high cholesterol group. The samples of the high and medium cholesterol groups from the progression phase clustered together. The top upregulated genes related to cell migration (*Kdr, Il1r1, Flt4, Adamts1, Sele, Aqp1, Sema6a, Hspb1, Sema3f*), angiogenesis (*Kdr, Flt4, Hspb1, Col4a1, Col4a2, Sema6a*) and extracellular matrix organization (*Lama4, Col4a1, Col4a2, Pxdn, Adamts1, Adamts4, Adamts7, Hmcn1*) (**Figure 5A**).

Considering the participation of macrophages in tissue immune homeostasis, we specifically looked at genes for cytokines that shifted their expression pattern after cholesterol reduction. Dietary cholesterol upregulated the expression of *Ccl2, Ccl3, Ccl4, Ccl17, Csf1, Cxcl2*, *Hbegf, Il10, Il1a, Il33, Tnf, Trail*, and *Vegfa*, which normalized after cholesterol reduction (**Figure 5B**). Additionally, we searched for the cytokines that were not “turned off” by cholesterol reduction and found that *Bmp2, Bmp6, Fgf1, Il27, Il6st*, and *Tgfa* remained upregulated in the high cholesterol group but normalized their expression in the medium cholesterol group (**Figure 5C**).

We examined the pathways modified by dietary cholesterol and detected the upregulation of TNF-α, TGF-β, and IL-6 signaling during the progression phase and the downregulation of cholesterol and fatty acid metabolisms, FC-γ receptor activation, and PD-1 signaling pathways. Cholesterol reduction normalized most of these pathways (**Figure 5D**). Notably, the high cholesterol group showed a non-reversible upregulation of collagen formation and extracellular matrix organization processes. Cholesterol intake enhanced the expression of several genes encoding fibrillar collagen, fibrillin, and basement membrane components during the progression phase of medium and high cholesterol groups (**Figure 5E**). While most of these genes normalized their expression after cholesterol reduction in the medium cholesterol group, several genes were still highly expressed in the high cholesterol group. Matrix and disintegrin metalloproteinases genes (MMPs and ADAMs, respectively) followed a similar expression pattern, except for *Mmp12, Mmp13*, and *Mmp19*, which were downregulated during the regression phase in both groups. *Timp3* was the only metalloproteinase inhibitor significantly expressed, displaying high levels in the medium and high cholesterol groups during the progression stage and the high cholesterol group in the regression phase.

**Figure 6:**
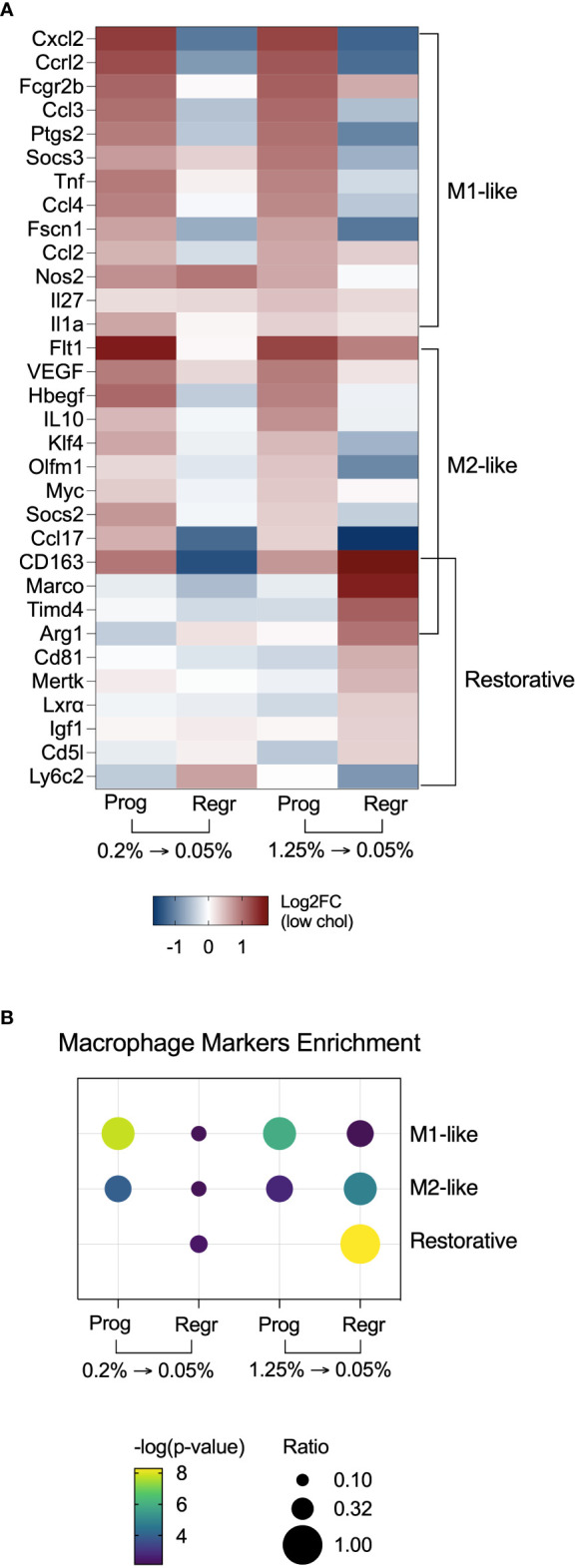
Hepatic macrophages from mice fed with high cholesterol FPC diet shift polarization towards a restorative phenotype after cholesterol reduction. **(A)** Heatmap with macrophage polarization markers shows enrichment of M1 and M2-like polarization markers during the progression of the FPC diet, and enrichment of restorative macrophages markers during the regression phase in the high cholesterol group. **(B)** Fisher’s exact test confirmed the enrichment of polarization markers in hepatic macrophages during the different phases of the dietary intervention. Prog, Progression phase; Regr, Regression phase; Log2FC, log2-foldchange based on low cholesterol age-matched group.

Noticing the upregulation of pro-inflammatory pathways, we decided to evaluate the expression of macrophage polarization markers in both dietary intervention phases (**Figure 6A**). We found that hepatic macrophages from both cholesterol groups expressed mixed M1 and M2 polarization markers during the progression phase. After cholesterol reduction, these markers lowered their expression. Surprisingly, macrophages from the high cholesterol group displayed a more significant enrichment of genes that characterize restorative macrophages after cholesterol reduction (**Figure 6B**). These findings suggest that high cholesterol intake may promote more substantial hepatic damage with a further expansion of the restorative macrophage population after cholesterol reduction.

Additionally, we analyzed the FACS sorting data for the presence of Kupffer cells (KCs: F4/80^hi^ CD11b^low^) and infiltrating macrophages (IMs: F4/80^low^ CD11b^hi^) to verify if the results we observed would relate to the infiltrating macrophage influx rates. We did not find any significant change in the IMs/KCs ratios among the different groups during the dietary intervention (**Supplementary Figure 3**), which suggests that the phenotypic changes detected in our dataset are not linked to the reduction of recruited macrophages.

### High cholesterol intake triggers NASH-associated pathways in hepatic macrophages

**Figure 7:**
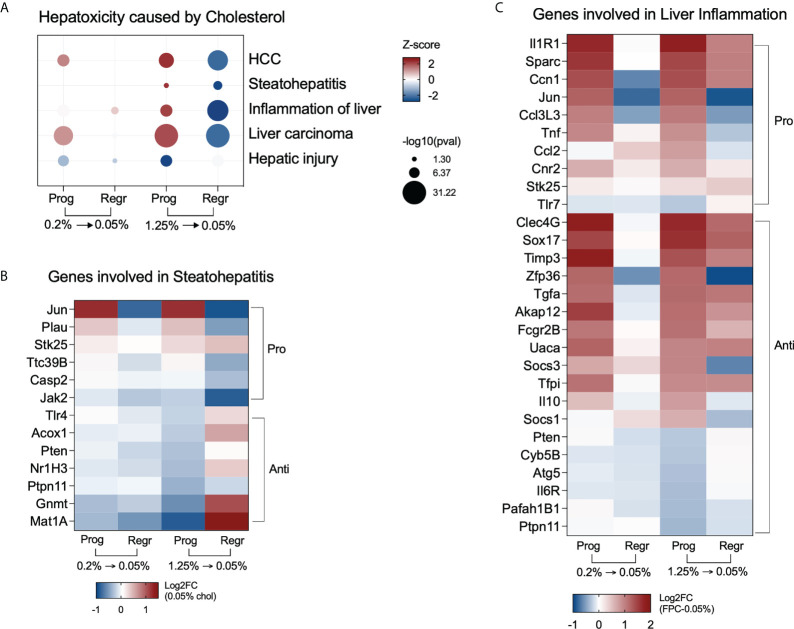
High cholesterol intake induces NASH-associated pathways in hepatic macrophages. **(A)** Hepatic macrophages from mice fed with high cholesterol displayed upregulation of steatohepatitis and liver inflammation processes during the progression phase and show inhibition of the same processes after cholesterol reduction. **(B)** Heatmap of the most variable genes involved in steatohepatitis in the high cholesterol group. During the progression phase, there is a strong downregulation of genes that can prevent NASH, such as Methionine adenosyltransferase (*Mat1*) and glycine N-methyltransferase (*Gnmt*), which were upregulated after cholesterol reduction. **(C)** Heatmap displaying the most variable genes related to liver inflammation in the high cholesterol group. Several pro-inflammatory genes triggered by high and medium cholesterol intake were normalized after cholesterol reduction, particularly *Jun, Ccl3l3*, *Tnf, Ccl2, and Cnr2*. The downregulation of the anti-inflammatory genes *Pten, Ptpn11, Cyb5b, Atg5, Il6r*, and *Pafah1b1* only in the high cholesterol group suggests their importance for NASH pathogenesis. Prog, Progression phase; Regr, Regression phase; Log2FC, log2-foldchange based on low cholesterol age-matched group.

We evaluated the most significant hepatotoxic pathways triggered in hepatic macrophages and identified upregulation of steatohepatitis, liver inflammation, and carcinoma in the high cholesterol group during the progression phase. Cholesterol reduction inhibited those processes (**Figure 7A**). **Figure 7B** shows the genes related to steatohepatitis significantly modified in macrophages of the high cholesterol group. We observed a shift in the expression pattern of anti-NASH genes, with upregulation of *Acox1, Nr1h3* (Lxr-a)*, Gnmt*, and *Mat1* expressions after cholesterol reduction. Genes controlling liver inflammation were also affected by cholesterol intake and following reduction (**Figure 7C**). The pro-inflammatory genes *Jun, Ccl3L3, Tnf*, and *Ccl2*, initially upregulated during the progression phase, were inhibited in the regression phase. The most notable change is related to the anti-inflammatory genes *Pten, Atg5, Il6r, Pafah1b1*, and *Ptpn11*, which presented a low gene expression due to cholesterol intake, with further normalization after cholesterol reduction.

## Discussion

Increasing evidence support the substantial contribution of innate and adaptive immunity in NASH progression. Among the innate immune cells, the hepatic macrophages play a pivotal role: they can cross-communicate with neutrophils, natural killer cells, innate lymphoid cells, and lymphocytes, coordinating cell recruitment, activation, and subsequent fate of hepatic inflammation (29–32).

By analyzing the impact of the dietary cholesterol reduction on NASH progression and on hepatic macrophage transcriptome, this work expands the understanding of the modulatory effects of cholesterol on the innate immune system, identifying potential new therapeutic targets for NASH intervention. High cholesterol intake combined with a high-fat diet contributes to hepatic lipid accumulation, liver oxidative stress, and consequent steatohepatitis development (6, 33). In our study, all animals on the FPC diet displayed high steatosis scores, regardless of cholesterol intake, indicating that cholesterol *per se* did not directly influence hepatic lipid accumulation. Instead, the cholesterol effect was related to tissue inflammation and scarring. Mice on high cholesterol diet displayed more lobular inflammation and fibrosis than mice fed with low cholesterol, corroborating our previous study (34). In the cholesterol reduction phase, we observed a slight decrease in lobular inflammation in the high cholesterol group, but fibrosis scores remained unchanged. The fact that we do not observe a reversion of fibrosis after reducing cholesterol may be due to the long-term high-fat and high-fructose diet that also culminates in fibrosis, even without cholesterol. The high cholesterol levels likely accelerate fibrosis progression. Alternatively, fibrosis may be irreversible at this stage.

Hepatic accumulation of free cholesterol during steatosis disturbs Kupffer cells and triggers a sterile inflammatory response (11, 35). Our findings demonstrate that prolonged medium or high cholesterol intake modifies total hepatic macrophage global gene expression. When we reduced cholesterol content in the FPC diet, the gene expression of macrophages from the medium and low cholesterol groups became very similar. However, cholesterol reduction did not normalize the gene expression in macrophages from mice fed high cholesterol diet, suggesting that the liver injury mediated by high cholesterol intake persists for longer. We also found that genes commonly affected by cholesterol intake and withdrawal, regardless of the initial cholesterol input, positively enriched biological processes linked to fibrosis during the progression stage and enhanced processes related to tissue repair during the regression phase. Of note, cholesterol upregulated the expression of some immunological genes in hepatic macrophages, which decreased after cholesterol reduction. Genes of interest include *Ccrl2*, an atypical chemokine receptor rapidly upregulated during inflammation (36); *Osm*, a cytokine that contributes to hepatic insulin resistance, fibrosis, development of NASH, and hepatocellular carcinoma (37–39); and *Ccl3*, a chemokine that favors the progression of steatohepatitis *via* macrophage recruitment (40).

Several pro-inflammatory and pro-fibrotic genes were exclusively induced in macrophages by medium and high cholesterol intake: *Osmr*, the receptor for Oncostatin M, is involved in macrophage recruitment and infiltration (41); *Il33*, a pro-inflammatory and pro-fibrotic cytokine that can activate hepatic stellate cells and macrophages (42, 43); *Tnf*, known to contribute to liver inflammation and strongly correlated to NASH severity (44); *Cnr2*, which participates in obesity-induced hepatic inflammation, macrophage infiltration, and TNF and CCL2 expression (45); and *Csf1*, highly expressed in NASH (46). Cholesterol reduction significantly inhibited the genes *Cxcl2* and *Ccl17*, known for neutrophil, macrophage, and Treg cell recruitment (47, 48). These findings support the significant impact of dietary cholesterol on macrophage-mediated hepatic inflammation in NASH.

We identified the most dramatic pathway changes in hepatic macrophage from mice fed with high cholesterol, with the upregulation of TNF-α, TGF-β, IFN-α, IL-6, and PDGF signaling pathways, all previously correlated to NASH progression. TNF-α signaling cross-regulates type I IFN signaling by eliciting interferon-stimulated gene expression and increasing inflammation, and IFN-α signaling would, in turn, favor steatosis (49, 50). IL-6 signaling also can contribute to NASH progression by increasing reactive oxygen species production and hepatocyte apoptosis (51). TGF-β and PDGF signaling pathways are well known to drive fibrosis progression, mainly by inducing hepatic stellate cell activation (52). In addition, these cells also upregulated steatohepatitis, inflammation, and cancer pathways during the progression phase, with their further silencing after cholesterol reduction.

Studies have shown that hypercholesterolemia impairs macrophage cholesterol efflux, driving their polarization into a pro-inflammatory phenotype (53). Our dataset showed a shift in macrophage polarization markers from the progression to the regression phase. Both medium and high cholesterol groups displayed M1 and M2-like markers enrichment during the progression phase. Intriguingly, macrophages from the high cholesterol group strongly expressed restorative macrophage markers after cholesterol reduction. Pro-restorative macrophages promote inflammation resolution and tissue repair, showing a distinct phenotype from the M1/M2 cells. Low expression of *Ly6C* and high expression of *Igf1, Cd81, Cd5l, Mertk*, and *Lxr* (54–56) characterize the pro-restorative phenotype. We also observed that the cholesterol intake or withdrawal does not influence the influx rates of infiltrating macrophages (IMs), suggesting that the polarization changes detected do not depend on the tissue ratios of KCs and IMs. Moreover, the increase of resident macrophages markers during the regression phase, such as *Cd163, Marco*, and *Timd4*, indicates that the restorative macrophage population expansion contributes to the repopulation of the resident macrophage pool during the late resolution stage of liver repair, as already implied by other authors (54). Upregulation of *Lxr* and *Mertk* gene expression also supports inflammation suppression since the *Lxr-Mertk* axis in macrophages promotes cholesterol efflux and efferocytosis (53). Our findings suggest that reducing high cholesterol intake is enough to establish a restorative macrophage subset in the liver, even on the background of high-fat and high-fructose diets.

Other remarkable events triggered by cholesterol intake in the hepatic macrophages were collagen formation and extracellular matrix organization, with the upregulation of genes codifying several types of collagens, including those part of the basement membrane, fibrillar collagen, and pro-fibrotic proteases. The increase of type IV collagen and laminin deposition on the perisinusoidal areas is a hallmark of fibrotic livers from alcoholic fatty liver, hepatitis, and cirrhosis patients (57). Since hepatic sinusoids typically lack a basement membrane, the increase of type IV collagen and laminin in the sinusoids would favor the formation of a new basement membrane. Basement membrane formation in the sinusoids is associated with liver sinusoidal endothelial cells (LSEC) defenestration, which precedes the activation of hepatic stellate cells and fibrosis (58). These events, however, were not normalized in macrophages from the high cholesterol groups during the regression phase. Therefore, the presence of macrophages expressing high levels of type IV collagen and laminin could continuously drive hepatic sinusoid capillarization and favor fibrosis progression even after the reduction of cholesterol.

Additional pro-fibrogenic factors continuously expressed by macrophages from the high cholesterol group were the *Il6st* (gp130), which regulates collagen and laminin expression; *Fgf1*, *Tgfa*, and *Pdgfd*, which activates hepatic stellate cells; and *Il13ra2*, which participates of TGF-β transcription (59–64). In addition, the expression of genes that favor steatosis, such as *Bmp2* and *Bmp6*, remained high after cholesterol reduction in this group (65, 66). Furthermore, hepatic macrophages from mice fed with high cholesterol diet did not restore the expression of the anti-fibrotic proteases *Mmp9*, *Mmp12*, and *Mmp13*, suggesting an aberrant tissue repair mechanism favoring fibrosis progression.

A limitation of our study is the use of RNAseq as the only method to evaluate the hepatic macrophages. We were able to use only this technique due to the small number of macrophages recovered from each mouse and the need to include the significant variations carried by the biological replicates. RNAseq is a valuable tool for quantifying the gene expression of the whole transcriptome. However, further studies should be performed to evaluate the functional capacity of hepatic macrophages in the different stages of NASH induced by cholesterol and validate all pathways described in our results.

In conclusion, our data shed light on the immunological mechanisms behind the contribution of dietary cholesterol to NASH progression. Here we demonstrated that cholesterol intake levels directly contribute to hepatic injury and that prolonged high cholesterol intake damages are long-lasting and persistent, further promoting the expansion of a dysfunctional pro-fibrotic hepatic restorative macrophage phenotype which continues even after cholesterol reduction.

## Data availability statement

The datasets presented in this study can be found in online repositories. The names of the repository/repositories and accession number(s) can be found below: https://www.ncbi.nlm.nih.gov/geo/query/acc.cgi?acc=GSE205776.

## Ethics statement

The animal study was reviewed and approved by University of Southern California Institutional Animal Care and Use Committee.

## Author contributions

AM-M and LG-M contributed to the conception and design of the study, analyzed the data, and wrote the manuscript. AM-M and AH executed the experiments. MS performed the bioinformatic analysis. GK performed the pathological evaluations. All authors contributed to manuscript revision, read, and approved the submitted version.

## Funding

This work was primarily supported by the National Institutes of Health grant numbers DK117004 & DK106491 (LG-M) and the USC Research Center for Liver Disease (P30DK048522).

## Acknowledgments

This manuscript is dedicated to the memory of Hugo R. Rosen, MD, who inspired a love for immunology and virology, and who inspired teamwork and respect for all. Dr. Rosen initiated and contributed significantly to this study. We would also like to thank Dr. Omar Lakhdari for performing the initial experiments.

## Conflict of interest

The authors declare that the research was conducted in the absence of any commercial or financial relationships that could be construed as a potential conflict of interest.

## Publisher’s note

All claims expressed in this article are solely those of the authors and do not necessarily represent those of their affiliated organizations, or those of the publisher, the editors and the reviewers. Any product that may be evaluated in this article, or claim that may be made by its manufacturer, is not guaranteed or endorsed by the publisher.

## References

[1] Neuschwander-Tetri BA. Hepatic lipotoxicity and the pathogenesis of nonalcoholic steatohepatitis: The central role of nontriglyceride fatty acid metabolites. Hepatology (2010) 52(2):774–88. doi: 10.1002/hep.23719 20683968

[2] Wree A Broderick L Canbay A Hoffman HM Feldstein AE. From NAFLD to NASH to cirrhosis-new insights into disease mechanisms. Nat Rev Gastroenterol Hepatol (2013) 10(11):627–36. doi: 10.1038/nrgastro.2013.149 23958599

[3] Younossi ZM Koenig AB Abdelatif D Fazel Y Henry L Wymer M. Global epidemiology of nonalcoholic fatty liver disease-meta-analytic assessment of prevalence, incidence, and outcomes. Hepatology (2016) 64(1):73–84. doi: 10.1002/hep.28431 26707365

[4] Puri P Baillie RA Wiest MM Mirshahi F Choudhury J Cheung O. A lipidomic analysis of nonalcoholic fatty liver disease. Hepatology (2007) 46(4):1081–90. doi: 10.1002/hep.21763 17654743

[5] Wouters K van Gorp PJ Bieghs V Gijbels MJ Duimel H Lütjohann D. Dietary cholesterol, rather than liver steatosis, leads to hepatic inflammation in hyperlipidemic mouse models of nonalcoholic steatohepatitis. Hepatology. (2008) 48(2):474–86. doi: 10.1002/hep.22363 18666236

[6] Bellanti F Villani R Facciorusso A Vendemiale G Serviddio G. Lipid oxidation products in the pathogenesis of non-alcoholic steatohepatitis. Free Radic Biol Med (2017) 111:173–85. doi: 10.1016/j.freeradbiomed.2017.01.023 28109892

[7] Püschel GP Henkel J. Dietary cholesterol does not break your heart but kills your liver. Porto BioMed J (2018) 3(1):e12. doi: 10.1016/j.pbj.0000000000000012 31595236 PMC6726297

[8] Marí M Caballero F Colell A Morales A Caballeria J Fernandez A. Mitochondrial free cholesterol loading sensitizes to TNF- and fas-mediated steatohepatitis. Cell Metab (2006) 4(3):185–98. doi: 10.1016/j.cmet.2006.07.006 16950136

[9] Ioannou GN Haigh WG Thorning D Savard C. Hepatic cholesterol crystals and crown-like structures distinguish NASH from simple steatosis. J Lipid Res (2013) 54(5):1326–34. doi: 10.1194/jlr.M034876 PMC3622327 23417738

[10] Tomita K Teratani T Suzuki T Shimizu M Sato H Narimatsu K. Free cholesterol accumulation in hepatic stellate cells: Mechanism of liver fibrosis aggravation in nonalcoholic steatohepatitis in mice. Hepatology. (2014) 59(1):154–69. doi: 10.1002/hep.26604 23832448

[11] Ioannou GN Subramanian S Chait A Haigh WG Yeh MM Farrell GC. Cholesterol crystallization within hepatocyte lipid droplets and its role in murine NASH. J Lipid Res (2017) 58(6):1067–79. doi: 10.1194/jlr.M072454 PMC5456359 28404639

[12] Devisscher L Verhelst X Colle I Van Vlierberghe H Geerts A. The role of macrophages in obesity-driven chronic liver disease. J Leukoc Biol (2016) 99(5):693–8. doi: 10.1189/jlb.5RU0116-016R 26936934

[13] Park JW Jeong G Kim SJ Kim MK Park SM. Predictors reflecting the pathological severity of non-alcoholic fatty liver disease: Comprehensive study of clinical and immunohistochemical findings in younger Asian patients. J Gastroenterol Hepatol (2007) 22(4):491–7. doi: 10.1111/j.1440-1746.2006.04758.x 17376039

[14] Han J Zhang X Lau JK Fu K Lau HC Xu W. Bone marrow-derived macrophage contributes to fibrosing steatohepatitis through activating hepatic stellate cells. J Pathol (2019) 248(4):488–500. doi: 10.1002/path.5275 30945293 PMC6767065

[15] Kazankov K Jørgensen SMD Thomsen KL Møller HJ Vilstrup H George J. The role of macrophages in nonalcoholic fatty liver disease and nonalcoholic steatohepatitis. Nat Rev Gastroenterol Hepatol (2019) 16(3):145–59. doi: 10.1038/s41575-018-0082-x 30482910

[16] Huang W Metlakunta A Dedousis N Zhang P Sipula I Dube JJ. Depletion of liver kupffer cells prevents the development of diet-induced hepatic steatosis and insulin resistance. Diabetes (2010) 59(2):347–57. doi: 10.2337/db09-0016 PMC2809951 19934001

[17] Im YR Hunter H de Gracia Hahn D Duret A Cheah Q Dong J. A systematic review of animal models of NAFLD finds high-fat, high-fructose diets most closely resemble human NAFLD. Hepatology (2021) 74(4):1884–901. doi: 10.1002/hep.31897 33973269

[18] Wang X Zheng Z Caviglia JM Corey KE Herfel TM Cai B. Hepatocyte TAZ/WWTR1 promotes inflammation and fibrosis in nonalcoholic steatohepatitis. Cell Metab (2016) 24(6):848–62. doi: 10.1016/j.cmet.2016.09.016 PMC5226184 28068223

[19] Akinrinmade OA Chetty S Daramola AK Islam MU Thepen T Barth S. CD64: An attractive immunotherapeutic target for M1-type macrophage mediated chronic inflammatory diseases. Biomedicines. (2017) 5(3):56. doi: 10.3390/biomedicines5030056 PMC5618314 28895912

[20] Cai B Dongiovanni P Corey KE Wang X Shmarakov IO Zheng Z. Macrophage MerTK promotes liver fibrosis in nonalcoholic steatohepatitis. Cell Metab (2020) 31(2):406–21.e7. doi: 10.1016/j.cmet.2019.11.013 31839486 PMC7004886

[21] Wang C Ma C Gong L Guo Y Fu K Zhang Y. Macrophage polarization and its role in liver disease. Front Immunol (2021) 12:803037. doi: 10.3389/fimmu.2021.803037 34970275 PMC8712501

[22] Patro R Duggal G Love MI Irizarry RA Kingsford C. Salmon provides fast and bias-aware quantification of transcript expression. Nat Methods (2017) 14(4):417–9. doi: 10.1038/nmeth.4197 PMC5600148 28263959

[23] Soneson C Love MI Robinson MD. Differential analyses for RNA-seq: transcript-level estimates improve gene-level inferences. F1000Res. (2015) 4:1521. doi: 10.12688/f1000research.7563.1 26925227 PMC4712774

[24] Love MI Huber W Anders S. Moderated estimation of fold change and dispersion for RNA-seq data with DESeq2. Genome Biol (2014) 15(12):550. doi: 10.1186/s13059-014-0550-8 25516281 PMC4302049

[25] Krämer A Green J Pollard J Tugendreich S. Causal analysis approaches in ingenuity pathway analysis. Bioinformatics. (2014) 30(4):523–30. doi: 10.1093/bioinformatics/btt703 PMC3928520 24336805

[26] Subramanian A Tamayo P Mootha VK Mukherjee S Ebert BL Gillette MA. Gene set enrichment analysis: A knowledge-based approach for interpreting genome-wide expression profiles. Proc Natl Acad Sci U S A. (2005) 102(43):15545–50. doi: 10.1073/pnas.0506580102 PMC1239896 16199517

[27] Mi H Muruganujan A Ebert D Huang X Thomas PD. PANTHER version 14: more genomes, a new PANTHER GO-slim and improvements in enrichment analysis tools. Nucleic Acids Res (2019) 47(D1):D419–D26. doi: 10.1093/nar/gky1038 PMC6323939 30407594

[28] Edgar R Domrachev M Lash AE. Gene expression omnibus: NCBI gene expression and hybridization array data repository. Nucleic Acids Res (2002) 30(1):207–10. doi: 10.1093/nar/30.1.207 PMC99122 11752295

[29] Li H Zhou Y Wang H Zhang M Qiu P Zhang R. Crosstalk between liver macrophages and surrounding cells in nonalcoholic steatohepatitis. Front Immunol (2020) 11:1169. doi: 10.3389/fimmu.2020.01169 32670278 PMC7326822

[30] Hirsova P Bamidele AO Wang H Povero D Revelo XS. Emerging roles of T cells in the pathogenesis of nonalcoholic steatohepatitis and hepatocellular carcinoma. Front Endocrinol (Lausanne) (2021) 12:760860. doi: 10.3389/fendo.2021.760860 34777255 PMC8581300

[31] Michel T Hentges F Zimmer J. Consequences of the crosstalk between monocytes/macrophages and natural killer cells. Front Immunol (2012) 3:403. doi: 10.3389/fimmu.2012.00403 23316194 PMC3539656

[32] Chen Y Tian Z. Roles of hepatic innate and innate-like lymphocytes in nonalcoholic steatohepatitis. Front Immunol (2020) 11:1500. doi: 10.3389/fimmu.2020.01500 32765518 PMC7378363

[33] Savard C Tartaglione EV Kuver R Haigh WG Farrell GC Subramanian S. Synergistic interaction of dietary cholesterol and dietary fat in inducing experimental steatohepatitis. Hepatology (2013) 57(1):81–92. doi: 10.1002/hep.25789 22508243 PMC5341743

[34] McGettigan B McMahan R Orlicky D Burchill M Danhorn T Francis P. Dietary lipids differentially shape nonalcoholic steatohepatitis progression and the transcriptome of kupffer cells and infiltrating macrophages. Hepatology (2019) 70(1):67–83. doi: 10.1002/hep.30401 30516830 PMC6923128

[35] Bieghs V Hendrikx T van Gorp PJ Verheyen F Guichot YD Walenbergh SM. The cholesterol derivative 27-hydroxycholesterol reduces steatohepatitis in mice. Gastroenterology (2013) 144(1):167–78.e1. doi: 10.1053/j.gastro.2012.09.062 23041327

[36] Del Prete A Martínez-Muñoz L Mazzon C Toffali L Sozio F Za L. The atypical receptor CCRL2 is required for CXCR2-dependent neutrophil recruitment and tissue damage. Blood (2017) 130(10):1223–34. doi: 10.1182/blood-2017-04-777680 28743719

[37] Henkel J Gärtner D Dorn C Hellerbrand C Schanze N Elz SR. Oncostatin m produced in kupffer cells in response to PGE2: Possible contributor to hepatic insulin resistance and steatosis. Lab Invest (2011) 91(7):1107–17. doi: 10.1038/labinvest.2011.47 21519329

[38] Foglia B Sutti S Pedicini D Cannito S Bocca C Maggiora M. A profibrogenic mediator overexpressed in non-alcoholic fatty liver disease, stimulates migration of hepatic myofibroblasts. Cells (2019) 9(1):28. doi: 10.3390/cells9010028 PMC7017087 31861914

[39] Di Maira G Foglia B Napione L Turato C Maggiora M Sutti S. Oncostatin m is overexpressed in NASH-related hepatocellular carcinoma and promotes cancer cell invasiveness and angiogenesis. J Pathol (2022) 257(1):82–95. doi: 10.1002/path.5871 35064579 PMC9315146

[40] Xu L Chen Y Nagashimada M Ni Y Zhuge F Chen G. CC chemokine ligand 3 deficiency ameliorates diet-induced steatohepatitis by regulating liver macrophage recruitment and M1/M2 status in mice. Metabolism (2021) 125:154914. doi: 10.1016/j.metabol.2021.154914 34656648

[41] Zhang X Li J Qin JJ Cheng WL Zhu X Gong FH. Oncostatin m receptor β deficiency attenuates atherogenesis by inhibiting JAK2/STAT3 signaling in macrophages. J Lipid Res (2017) 58(5):895–906. doi: 10.1194/jlr.M074112 28258089 PMC5408608

[42] Tan Z Liu Q Jiang R Lv L Shoto SS Maillet I. Interleukin-33 drives hepatic fibrosis through activation of hepatic stellate cells. Cell Mol Immunol (2018) 15(4):388–98. doi: 10.1038/cmi.2016.63 PMC6052839 28194023

[43] Gao Y Liu Y Yang M Guo X Zhang M Li H. IL-33 treatment attenuated diet-induced hepatic steatosis but aggravated hepatic fibrosis. Oncotarget. (2016) 7(23):33649–61. doi: 10.18632/oncotarget.9259 PMC5085109 27172901

[44] Kakino S Ohki T Nakayama H Yuan X Otabe S Hashinaga T. Pivotal role of TNF-α in the development and progression of nonalcoholic fatty liver disease in a murine model. Horm Metab Res (2018) 50(1):80–7. doi: 10.1055/s-0043-118666 28922680

[45] Deveaux V Cadoudal T Ichigotani Y Teixeira-Clerc F Louvet A Manin S. Cannabinoid CB2 receptor potentiates obesity-associated inflammation, insulin resistance and hepatic steatosis. PloS One (2009) 4(6):e5844. doi: 10.1371/journal.pone.0005844 19513120 PMC2688760

[46] Fourman LT Stanley TL Billingsley JM Sui SJH Feldpausch MN Boutin A. Delineating tesamorelin response pathways in HIV-associated NAFLD using a targeted proteomic and transcriptomic approach. Sci Rep (2021) 11(1):10485. doi: 10.1038/s41598-021-89966-y 34006921 PMC8131688

[47] Moles A Murphy L Wilson CL Chakraborty JB Fox C Park EJ. A TLR2/S100A9/CXCL-2 signaling network is necessary for neutrophil recruitment in acute and chronic liver injury in the mouse. J Hepatol (2014) 60(4):782–91. doi: 10.1016/j.jhep.2013.12.005 PMC3960359 24333183

[48] Zhou SL Zhou ZJ Hu ZQ Huang XW Wang Z Chen EB. Tumor-associated neutrophils recruit macrophages and T-regulatory cells to promote progression of hepatocellular carcinoma and resistance to sorafenib. Gastroenterology (2016) 150(7):1646–58.e17. doi: 10.1053/j.gastro.2016.02.040 26924089

[49] Gordon RA Grigoriev G Lee A Kalliolias GD Ivashkiv LB. The interferon signature and STAT1 expression in rheumatoid arthritis synovial fluid macrophages are induced by tumor necrosis factor α and counter-regulated by the synovial fluid microenvironment. Arthritis Rheumatol (2012) 64(10):3119–28. doi: 10.1002/art.34544 PMC3449023 22614743

[50] Møhlenberg M Terczynska-Dyla E Thomsen KL George J Eslam M Grønbæk H. The role of IFN in the development of NAFLD and NASH. Cytokine. (2019) 124:154519. doi: 10.1016/j.cyto.2018.08.013 30139548

[51] Yamaguchi K Itoh Y Yokomizo C Nishimura T Niimi T Fujii H. Blockade of interleukin-6 signaling enhances hepatic steatosis but improves liver injury in methionine choline-deficient diet-fed mice. Lab Invest (2010) 90(8):1169–78. doi: 10.1038/labinvest.2010.75 20368703

[52] Barreby E Chen P Aouadi M. Macrophage functional diversity in NAFLD - more than inflammation. Nat Rev Endocrinol (2022) 18:461–72. doi: 10.1038/s41574-022-00675-6 35534573

[53] Tall AR Yvan-Charvet L. Cholesterol, inflammation and innate immunity. Nat Rev Immunol (2015) 15(2):104–16. doi: 10.1038/nri3793 PMC4669071 25614320

[54] Ramachandran P Pellicoro A Vernon MA Boulter L Aucott RL Ali A. Differential ly-6C expression identifies the recruited macrophage phenotype, which orchestrates the regression of murine liver fibrosis. Proc Natl Acad Sci USA (2012) 109(46):E3186–95. doi: 10.1073/pnas.1119964109 PMC3503234 23100531

[55] Triantafyllou E Pop OT Possamai LA Wilhelm A Liaskou E Singanayagam A. MerTK expressing hepatic macrophages promote the resolution of inflammation in acute liver failure. Gut (2018) 67(2):333–47. doi: 10.1136/gutjnl-2016-313615 PMC5868289 28450389

[56] Choi JY Seo JY Yoon YS Lee YJ Kim HS Kang JL. Mer signaling increases the abundance of the transcription factor LXR to promote the resolution of acute sterile inflammation. Sci Signal (2015) 8(365):ra21. doi: 10.1126/scisignal.2005864 25714463

[57] Mak KM Mei R. Basement membrane type IV collagen and laminin: An overview of their biology and value as fibrosis biomarkers of liver disease. Anat Rec (Hoboken) (2017) 300(8):1371–90. doi: 10.1002/ar.23567 28187500

[58] Martinez-Hernandez A Martinez J. The role of capillarization in hepatic failure: studies in carbon tetrachloride-induced cirrhosis. Hepatology (1991) 14(5):864–74. doi: 10.1002/hep.1840140519 1718835

[59] Huang H Zhang G Ge Z. lncRNA MALAT1 promotes renal fibrosis in diabetic nephropathy by targeting the miR-2355-3p/IL6ST axis. Front Pharmacol (2021) 12:647650. doi: 10.3389/fphar.2021.647650 33995063 PMC8117091

[60] Yu C Wang F Jin C Huang X Miller DL Basilico C. Role of fibroblast growth factor type 1 and 2 in carbon tetrachloride-induced hepatic injury and fibrogenesis. Am J Pathol (2003) 163(4):1653–62. doi: 10.1016/S0002-9440(10)63522-5 PMC1868310 14507672

[61] Wang C Li Y Li H Zhang Y Ying Z Wang X. Disruption of FGF signaling ameliorates inflammatory response in hepatic stellate cells. Front Cell Dev Biol (2020) 8:601. doi: 10.3389/fcell.2020.00601 32793588 PMC7387415

[62] Lee KS Buck M Houglum K Chojkier M. Activation of hepatic stellate cells by TGF alpha and collagen type I is mediated by oxidative stress through c-myb expression. J Clin Invest (1995) 96(5):2461–8. doi: 10.1172/JCI118304 PMC185899 7593635

[63] Borkham-Kamphorst E Meurer SK Van de Leur E Haas U Tihaa L Weiskirchen R. PDGF-d signaling in portal myofibroblasts and hepatic stellate cells proves identical to PDGF-b *via* both PDGF receptor type α and β. Cell Signal (2015) 27(7):1305–14. doi: 10.1016/j.cellsig.2015.03.012 25819339

[64] Fichtner-Feigl S Strober W Kawakami K Puri RK Kitani A. IL-13 signaling through the IL-13alpha2 receptor is involved in induction of TGF-beta1 production and fibrosis. Nat Med (2006) 12(1):99–106. doi: 10.1038/nm1332 16327802

[65] Thayer TE Lino Cardenas CL Martyn T Nicholson CJ Traeger L Wunderer F. The role of bone morphogenetic protein signaling in non-alcoholic fatty liver disease. Sci Rep (2020) 10(1):9831. doi: 10.1038/s41598-020-66770-8 32561790 PMC7305229

[66] Arndt S Wacker E Dorn C Koch A Saugspier M Thasler WE. Enhanced expression of BMP6 inhibits hepatic fibrosis in non-alcoholic fatty liver disease. Gut. (2015) 64(6):973–81. doi: 10.1136/gutjnl-2014-306968 25011936

