# Supplementary material for: Hepatic damage caused by long-term high cholesterol intake induces a dysfunctional restorative macrophage population in experimental NASH
Source: Front Immunol. 2022 Sep 8;13:968366. doi: 10.3389/fimmu.2022.968366 (PMC9495937; doi:10.3389/fimmu.2022.968366)
Supplement: Supplementary file 1 [file DataSheet_1.docx]

Hepatic damage caused by long-term high cholesterol intake induces a dysfunctional restorative macrophage population in experimental NASH.

Ana Maretti-Mira, Matthew P. Salomon, Angela M. Hsu, Chikako Matsuba, Gary C. Kanel, Lucy Golden-Mason

Contents List:

Supplementary Figure 1

Supplementary Figure 2

Supplementary Figure 3


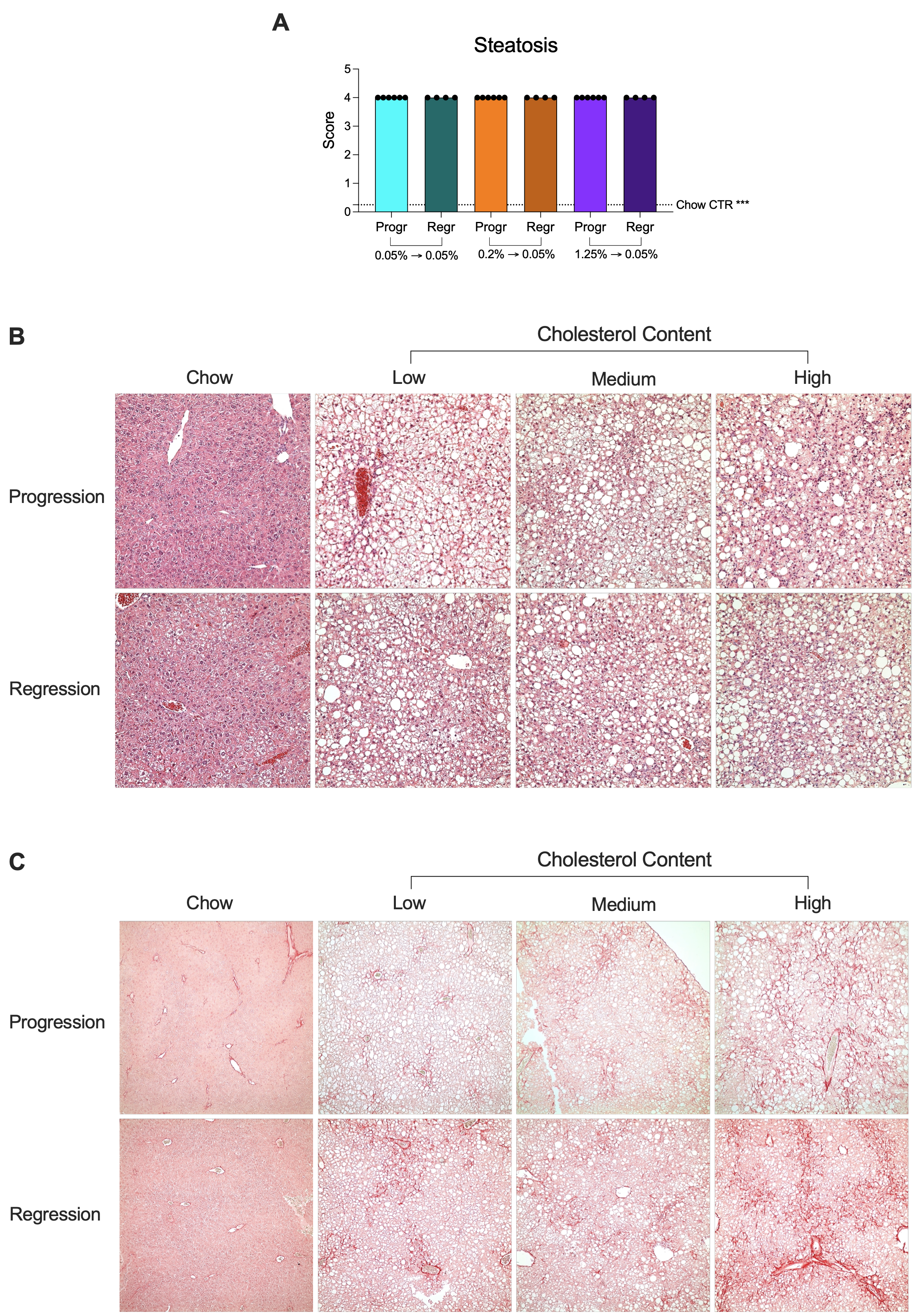


**Supplementary Figure 1:** Histological findings. (A) Steatosis score. All groups showed similar steatosis grade regardless of cholesterol intake. Prog: Progression phase; Regr: Regression phase; CTR: control. (B) Representative images of histological hematoxylin-eosin staining. Magnification 200x. Statistical analysis of Inflammation Scores shown on Figure 1A. (C) Representative images of histological Sirius Red staining. Magnification 100x. Statistical analysis of Fibrosis Scores shown on Figure 1B.


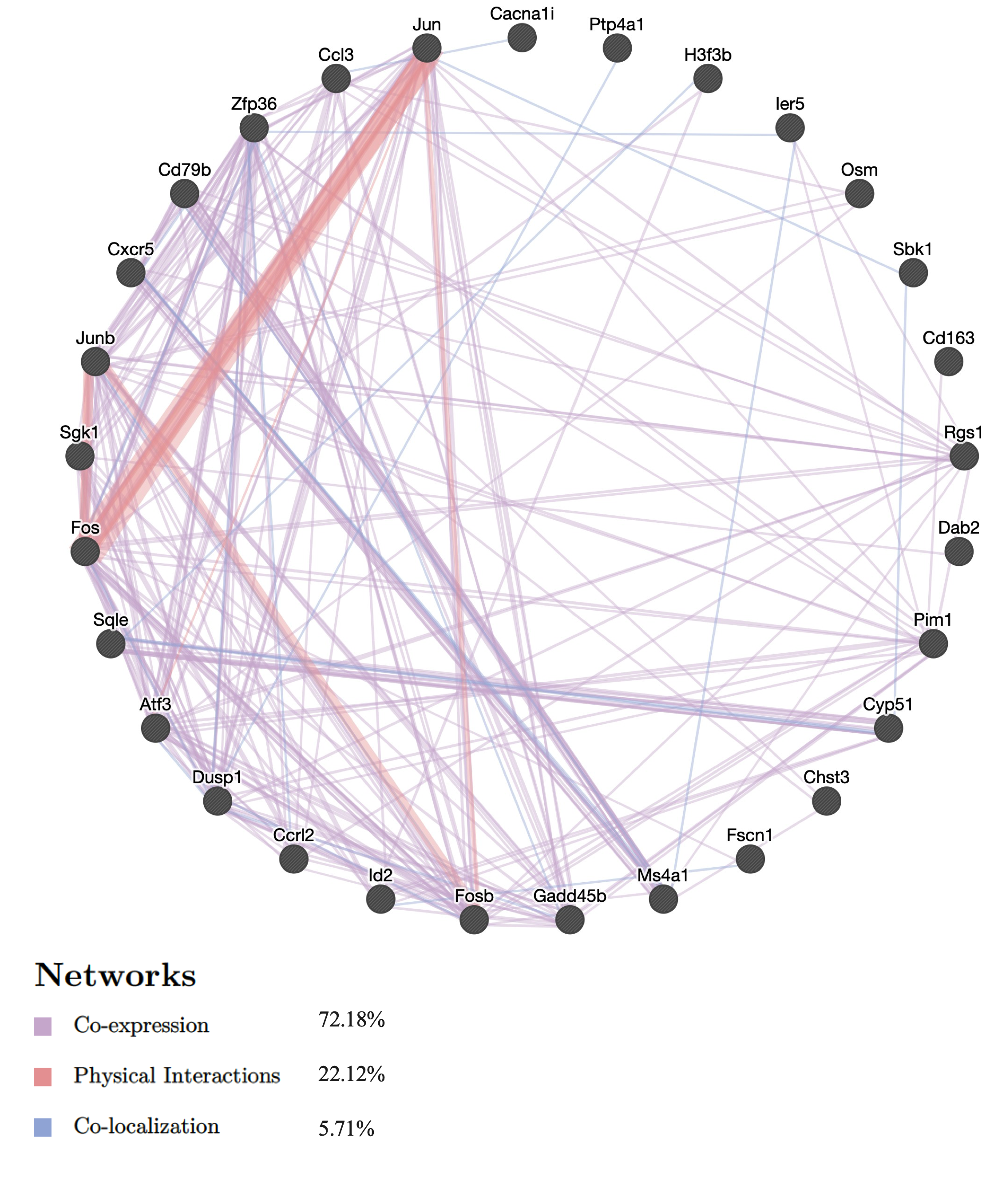


**Supplementary Figure 2:** Network strength among the genes commonly induced dietary cholesterol. GeneMANIA network analysis test showed that the 30 of the 32 genes commonly modified by dietary cholesterol are highly co-expressed. Physical interaction and co-localization were also indicated by the analysis.


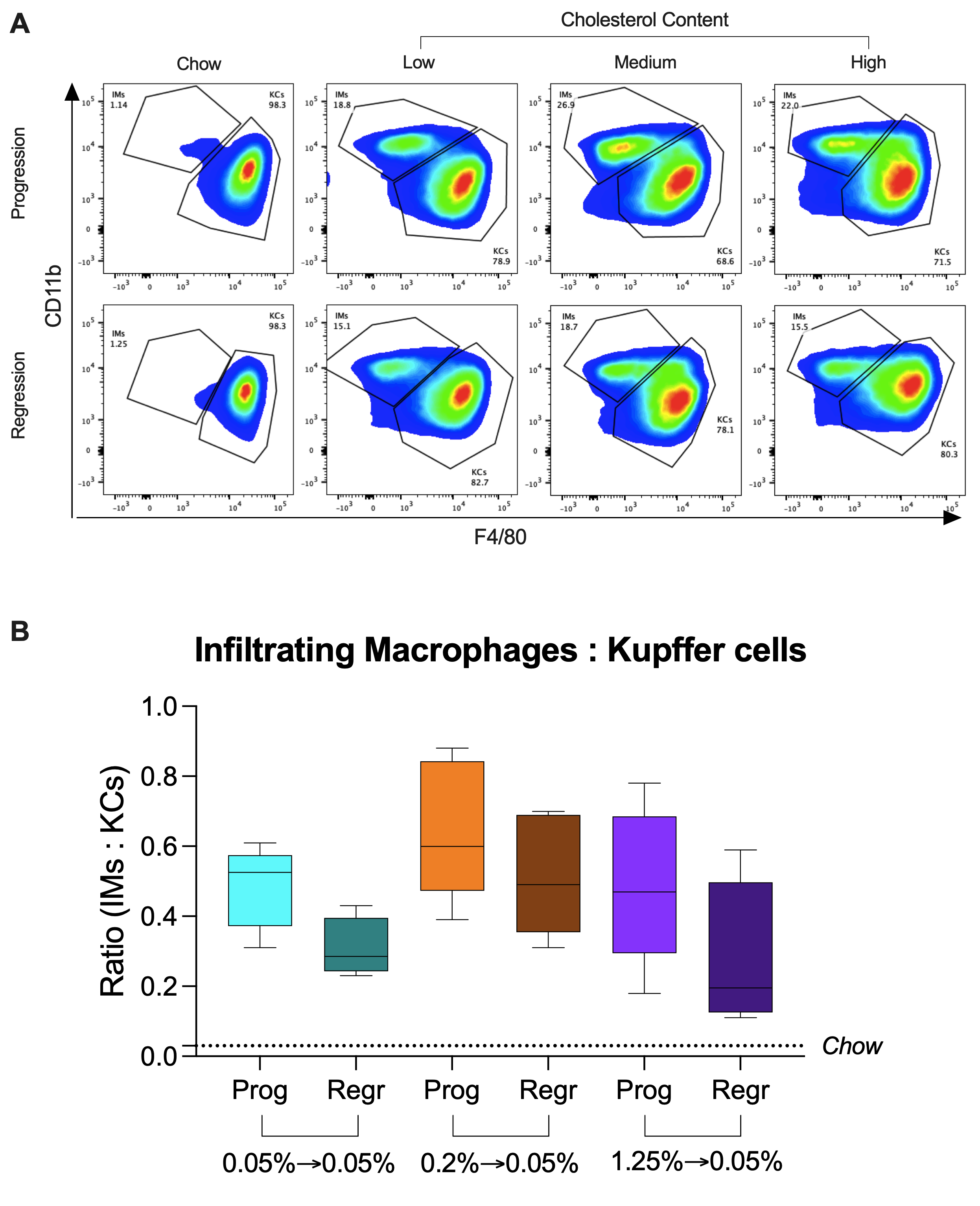


**Supplementary Figure 3:** Frequency of hepatic macrophage subpopulations. (A) Representative graphics from FACS sorting showing Kupffer cells (KCs – F480^hi^ CD11b^low^) and Infiltrating macrophages (IMs - F480^low^ CD11b^hi^). (B) Ratios of IMs: KCs. We calculated the total hepatic macrophages by summing the total number of IMs and KCs. Then, we defined the real KCs and IMs subpopulation percentages. Ratios were calculated by dividing IM by KC frequencies. No statistical differences were found in the IM: KC ratios during the dietary intervention. The statistical test applied One-Way ANOVA followed by Tukey's multiple comparisons test. Prog: Progression phase; Regr: Regression phase; CTR: control.
